# Ursodeoxycholic Acid and SMOFlipid for Treating Parenteral Nutrition Associated Cholestasis in Infants

**DOI:** 10.7759/cureus.22060

**Published:** 2022-02-09

**Authors:** Saleh Al-Alaiyan, Weam Elsaidawi, Amal M Alanazi, Raef A Qeretli, Najlaa A Abdulaziz, Areej Alfattani

**Affiliations:** 1 Pediatrics/Neonatal-Perinatal Medicine, King Faisal Specialist Hospital & Research Centre, Riyadh, SAU; 2 Pediatrics, Alfaisal University, Riyadh, SAU; 3 Pediatrics, King Faisal Specialist Hospital & Research Centre, Riyadh, SAU; 4 Pharmacology and Therapeutics, King Faisal Specialist Hospital & Research Centre, Riyadh, SAU; 5 Biostatistics and Epidemiology, King Faisal Specialist Hospital & Research Centre, Riyadh, SAU

**Keywords:** total parenteral nutrition, smoflipid, ursodiol, cholestasis, neonates

## Abstract

Background: Parenteral nutrition-associated cholestasis (PNAC) is frequently seen in preterm infants receiving total parenteral nutrition (TPN) for a long duration. The pathogenesis of PNAC is believed to be multifactorial; however, phytosterols are hepatotoxic, resulting in cholestasis. A novel lipid emulsion consisting of a mixture of soybean oil, medium-chain triglycerides, olive oil, and fish oil (SMOFlipid) with a low level of phytosterols has been shown to improve cholestasis. Moreover, ursodeoxycholic acid (UDCA) has improved bile flow and normalized liver function tests. This study aimed to determine the effect of UDCA and SMOFlipid in preventing and treating PNAC in infants.

Methods: We conducted a retrospective cohort study that included all infants who received TPN for at least five days between January 2010 and December 2018, who also received UDCA for the treatment of cholestasis, and infants who developed cholestasis but were not treated with UDCA. In addition, any infants who received SMOFlipid for parenteral nutrition during the same period were included. We recorded multiple variables, including neonatal demographic data, major medical diagnosis, liver function, medications, and maternal variables.

Results: A total of 58 infants with cholestasis who received UDCA for treatment were identified. The infants were divided into two groups, Group 1 infants had gestational age (GA) of ≤32 weeks, and Group 2 had GA of >32 weeks. We found that combining SMOFlipid with UDCA resulted in a significant reduction in cholestasis duration in both groups. Infants in Group 1 who received SMOFlipid had cholestasis for a mean of 67 ± 57 days, and those who did not receive SMOFlipid had cholestasis for a mean of 145 ± 102 days (p=0.04). Infants in Group 2 who received SMOFlipid had cholestasis for a mean of 38.2 ± 28 days, and those who did not receive SMOFlipid had cholestasis for a mean of 117 ± 119 days (p=0.02).

Conclusions: According to our results, the use of UDCA and SMOFlipid reduced the duration of parenteral nutrition-associated with cholestasis in very low birth weight infants.

## Introduction

Parenteral nutrition-associated cholestasis (PNAC) is often seen in preterm infants receiving prolonged total parenteral nutrition (TPN). Bile is mainly composed of bile acids, bilirubin, and fats. It is formed in the liver and secreted into the canaliculus, then flows into biliary ducts from where it is eventually secreted into the intestine after temporary storage within the gallbladder. Hepatic cholestasis usually results from an impaired balance between bile acid uptake and efflux. This impairment leads to abnormal hepatic accumulation of bile salts that disrupt cell membranes and cellular organelles, then hepatic cell necrosis, inflammation, and fibrosis occur [1]. In clinical practice, it is characterized by direct jaundice and/or increased levels of hepatic biomarkers such as gamma-glutamyl transpeptidase (GGT) and/or alkaline phosphatase (ALP) [2]. Prolonged bowel rest facilitates intestinal bacterial overgrowth and causes decreased cholecystokinin secretion in the duodenum leading to diminished gallbladder contractility and bile stasis [3]. Cholestatic jaundice affects approximately one in 2,500 infants and has multiple etiologies [4]. Approximately 18% to 67% of infants who receive TPN for more than two weeks are at risk of developing hepatic injury and cholestasis [5]. The incidence of PNAC increases as the duration of TPN increases. In a study that included 1,366 infants, neonates who received TPN for 14 to 28 days had a 14% incidence of PNAC, those who received TPN for 29 to 56 days had a 43% incidence, those who received TPN for 57 to 100 days had a 72% incidence, and those who received TPN for >100 days had an 85% incidence of PNAC [6]. Infants at high risk of developing PNAC can be identified early in their hospital course. Other risk factors for cholestatic jaundice are extremely low birth weight in infants and those with congenital gastrointestinal anomalies such as gastroschises and jejunal atresia [6].

The pathogenesis of PNAC is believed to be multifactorial; however, the soybean-based lipid emulsion component of TPN has been flagged as a potential contributing factor in PNAC. Soybean-based emulsions are rich in omega-6 fatty acids, which have pro-inflammatory products that negatively affect immunologic function. They contain phytosterols, a group of plant steroid compounds that have been reported to be toxic to the liver by decreasing bile flow in animal studies [7-9] and can contribute to parenteral nutrition-associated cholestasis. Several trials had found a reversal of PNAC when standard soy-based parenteral lipid emulsion was replaced with fish oil-based lipid emulsion [10-12]. According to Mahmud et al., a novel lipid emulsion “containing a mixture of soybean oil, medium-chain triglycerides, olive oil, and fish oil (SMOFlipid®) with reduced omega-6 fatty acids, increased omega-3 fatty acids, and enriched in vitamin E was found to decrease the GGT serum level” [13].

The complications of long-standing cholestasis can be fatal. End-stage liver disease may develop in 3% to 15% of affected infants. In these severely affected infants, the mortality rate is very high without proper therapeutic interventions [6].

Infants receiving TPN should start enteral feedings as early as possible to stimulate bile flow, gallbladder contraction, and intestinal motility. Extreme low birth weight infants will benefit from trophic feeds that have been beneficial in reducing the incidence and severity of PNAC. Several medications have been used to reduce the level of conjugated bilirubin, with variable results such as phenobarbitone, erythromycin, and ursodeoxycholic acid (UDCA). Studies in animals with induced PNAC showed that UDCA improves bile flow and normalizes liver function tests [14]. In another study, adding metronidazole to UDCA therapy further decreased serum bilirubin concentrations and prevented parenteral nutrition-associated liver histological abnormalities [15]. Therefore, this study aimed to determine the effect of UDCA and SMOFlipid for the prevention and treatment of PNAC in infants.

## Materials and methods

We conducted a retrospective cohort study that included all infants who received TPN for at least five days between January 2010 and December 2018, who received UDCA for the treatment of cholestasis, and infants who developed cholestasis but were not treated with UDCA. UDCA was given as per the approved protocol in our hospital as 15 to 30 mg/kg/day orally in divided doses. In addition, infants who received SMOFlipid for parenteral nutrition during the same period were included. We collected infant demographic data, including gestational age (GA), gender, birth weight, and Apgar score. Major medical diagnoses include necrotizing enterocolitis (NEC), bronchopulmonary dysplasia, intraventricular hemorrhage (IVH; grade > 3), periventricular leukomalacia, and retinopathy of prematurity were collected. Drug data, including daily dose and length of therapy, were collected from the medical records of eligible infants. We collected maternal age, gravidity, parity, and abortions. Cholestasis data, including time from onset of cholestasis to treatment, peak direct bilirubin, duration of cholestasis, and dose of UDCA used for the infant, were also recorded. We also noted clinical data including length of TPN in days, trend of GGT, ALP, conjugated bilirubin, aspartate transaminase (AST), alanine transaminase (ALT), and albumin blood level before and during the use of UDCA.

Infants were excluded from the study if they tested positive for Toxoplasma gondii, rubella, cytomegalovirus, and herpes simplex virus (i.e., TORCH), had evidence of congenital biliary tract anomalies, major malformation, congenital hypothyroidism, or in-born errors of metabolism that resulted in cholestasis such as galactosemia, phenylketonuria, and antitrypsin-1 deficiency.

Definitions

Conjugated hyperbilirubinemia was defined as a serum conjugated bilirubin concentration greater than 1.0 mg/dL (17.1 µmol/L) if the total bilirubin is <5.0 mg/dL (85.5 µmol/L) or >20% of the total bilirubin if the total bilirubin is >5.0 mg/dL (85.5 µmol/L). Bronchopulmonary dysplasia (BPD) was diagnosed according to the National Institute of Child Health and Human development criteria [16]. NEC grade ≥ 2A was diagnosed and classified according to the modified Bell criteria [17]. IVH was diagnosed if patients’ hemorrhaging was greater than Grade 3 on Papile’s classification of grade > 3 [18]. Retinopathy of prematurity (ROP) was diagnosed and graded according to the International Classification of Retinopathy of Prematurity [19]. Cystic periventricular leukomalacia (PVL) was diagnosed with hypoechoic cysts in the periventricular white matter [20].

Statistical analysis

After data management and coding, we used SAS Software Version 9.4 (SAS Institute, Cary, NC) for statistical analysis. Descriptive statistics were calculated. We used the Wilcoxon Rank Sum test for continuous variables and Fisher's Exact test for categorical variables. We considered p<0.05 as statistically significant.

## Results

A total of 58 infants had cholestasis and were treated with UDCA. These infants were divided into two groups according to gestational age (GA); Group 1 (n=26) consisted of infants with GA ≤32 weeks, and Group 2 (n=32) consisted of infants with GA >32 weeks. One infant in Group 2 was positive for TORCH and was excluded from the study.

As expected and due to very low birth weight, there was a statistically significant difference between Group 1 and Group 2 (Group 1 mean birth weight, 1136.2 ± 654 g; Group 2 mean birth weight, 2782.3 ± 784 g; p=0.01). Group 1 infants' mean GA was 28.1 ± 2.8 weeks, and Group 2 infants' mean GA was 36.1 ± 1.5 weeks (p=0.01). We noted statistically significantly higher rates of ROP (p=0.01), NEC (p=0.01), and IVH (p=0.04) in Group 1 than Group 2. There were no statistically significant differences in the rate of BPD and PVL between the two groups. Rhesus isoimmunization was found in one infant in Group 1 and eight infants in Group 2 (p=0.031; Table 1).

**Table 1:** Demographic data and complications of prematurity in the two groups GA: gestational age; BPD: bronchopulmonary dysplasia; ROP: retinopathy of prematurity; NEC: necrotizing enterocolitis; IVH: intraventricular hemorrhage; PVL: periventricular leukomalacia; Rh: rhesus.

	Group 1 (≤ 32 weeks GA; n=26)	Group 2 (>32 weeks GA; n=32)	P-Value
Birth weight (g), mean ± SD	1136.2 ± 654	2782.3 ± 784	0.01
Gestational age (days), mean ± SD	28.1 ± 2.8	36.1 ± 1.5	0.01
Apgar score, mean ± SD	4 ± 2.2	4 ± 2.6	0.77
Maternal age (years), mean ± SD	28 ± 8.1	30 ± 5.6	0.3
BPD, n (%)	6 (23.1%)	4 (12.9%)	0.48
ROP, n (%)	13 (50%)	0 (0%)	0.000
NEC, n (%)	9 (34.6%)	2 (6.5%)	0.016
IVH, n (%)	8 (32%)	3 (9.7%)	0.048
PVL, n (%)	4 (15.4%)	1 (3.2%)	0.167
Rh isoimmunization, n (%)	1 (3.8%)	8 (25.8%)	0.031

In Table 2, despite the high levels, there were no significant statistical differences between the two groups in peak bilirubin level, ALT, AST, and GGT levels. The alkaline phosphatase was significantly higher in Group 1 than Group 2 (p<0.01). There were no statistically significant differences in the median age of infants between Group 1 (60 to 123 days) and Group 2 (40 to 104 days) at which UDCA was started (p=0.382). There was a delay in starting UDCA in both groups despite cholestasis. Regardless of the duration of cholestasis in both groups, the mean age of improvement after starting UDCA was almost the same (Group 1, 19.56 ± 5.1 days; Group 2, 20 ± 3.8 days; p=0.90).

**Table 2:** Cholestatic, UDCA and liver function variables in the two groups UDCA: ursodeoxycholic acid; TORCH: Toxoplasma gondii, other agents, rubella, cytomegalovirus, and herpes simplex virus; ALT: alanine transaminase; AST: aspartate transaminase; GGT: gamma-glutamyl transpeptidase.

	Group 1 (≤ 32 weeks GA; n=26)	Group 2 (>32 weeks GA; n=32)	P-Value
TORCH	0 (0%)	1 (3.1%)	1.000
Peak bilirubin (when UDCA started; μmol/L), mean ± SD	261 ± 26	282.3 ± 29	0.603
ALT (IU/L), mean ± SD	246 ±27	379 ± 131	0.357
AST (IU/L), mean ± SD	421 ± 99	635 ± 184	0.33
ALP (IU/L), mean ± SD	884 ± 75	508 ± 43	< 0.01
GGT (IU/L), mean ± SD	192 ± 45	268 ± 62	0.361
Duration of treatment (days), mean ± SD	81.6 ± 5.9	40 ± 37	< 0.01
Cholestasis improved (days), range	8 to 30	12 to 28	0.9
Age of infants when UDCA started (days), range	60 to 123	40 to 104	0.382

Combining SMOFlipid with UDCA showed a significant reduction in the duration of cholestasis in the two groups who received SMOFlipid. In Group 1, the duration of cholestasis with SMOFlipid was 67 ± 57 days and 145 ± 102 days without SMOFlipid (p=0.04). In Group 2, the duration of cholestasis with SMOFlipid was 38.2 ± 28 days and 117 ± 119 days without SMOFlipid (p=0.02).

## Discussion

In this retrospective cohort study, we found that combining SMOFlipid with UDCA led to a significant reduction in the duration of cholestasis in both groups. Increasing evidence suggests that SMOFlipid can improve PNAC. A meta-analysis of seven studies consisted of three studies (93 participants) whose primary outcome was the reversal of PNAC and four studies (1012 participants) designed to prevent PNAC. Authors found that the use of SMOFlipid was more likely to reverse PNAC, but it did not have a significant effect in preventing PNAC [21].

The mechanism by which SMOFlipid prevents PNAC is unknown. It has been postulated that a component of soy oil lipid emulsions in parenteral nutrition solutions, such as plant sterols (phytosterols), may be accountable for PNAC. In contrast, the use of SMOFlipid may be protective because it has lower phytosterol content than soy oil lipid emulsions [22]. In an animal study, the authors found that SMOFlipid restored bile acid flow into the gut, where it shaped the gut microbiota and metabolome profile more than with soybean oil emulsions [23]. They concluded that uninterrupted bile flow induced by SMOFlipid probably employs a dominant effect in reducing bile acid-sensitive Gram-positive bacteria via a shift toward a greater relative abundance of Gram-negative Enterobacteriaceae. These Gram-positive bacteria are responsible for increasing cholestasis by deconjugating the conjugated bile acids in the gut. While UDCA is proven to be effective in treating parenteral nutrition-associated liver disease (PNALD) in small studies, it should not be used as a preventive measure in high-risk infants [24-26].

In this study, we found a delay in starting UDCA in infants diagnosed with cholestasis. Although there were no statistical differences between the two groups, the delay in starting UDCA in very low birth weight infants (i.e., ≤ 32 weeks' gestation) was longer than four months. Moreover, the duration of treatment was longer among very low birth weight infants, but the time to response in both groups to UDCA was similar (eight to 30 days in Group 1; 12 to 28 in Group 2; p=0.90).

In contrast, a study that compared premature infants receiving soybean oil-based injectable lipid emulsion with infants who received SMOFlipid found no statistically significant difference in the development of cholestasis [27]. Similar to our findings, infants in that study who received ursodiol in the SMOFlipid group required fewer days of therapy than those who received ursodiol in the soybean oil-based group.

Other risk factors for neonatal cholestasis, such as cytomegalovirus infection, genetic/metabolic diseases, hemolytic diseases, and biliary atresia, should be identified for determining the prognosis and for proper intervention. In this study, eight infants (>32 weeks' gestation; 25.8%) were diagnosed with hemolytic disease of the newborn (HDN) due to Rhesus isoimmunization. As a result of the HDN, cholestasis was expected to be prolonged and severe in some cases; therefore, parents were informed of the prognosis and the need for close follow-up [28]. The cause of cholestasis in neonates with HDN has been attributed to iron overload due to intrauterine transfusions [29].

UDCA has been more effective than phenobarbital in treating PNALD. In a retrospective cohort study that included infants with cholestasis, authors found that phenobarbital had limited efficacy in reducing conjugated bilirubin compared to UDCA [30]. Despite the limited evidence of its efficacy, neonatal intensive care providers still use phenobarbital to treat PNALD.

Our study was not without limitations. The study design was retrospective and used a small sample size. In addition, the method of collecting data may have unintentionally led to the exclusion of certain important information.

## Conclusions

PNAC is a well-recognized adverse effect of prolonged use of TPN in premature infants. The use of UDCA and SMOFlipid reduced the duration of PNAC in very low birth weight infants. This study also provides the basis for more extensive future prospective studies to improve the clinical outcome of infants with cholestasis.
